# Migrants' living conditions, perceived health needs and implications for the use of antibiotics and antimicrobial resistance in the United Kingdom: A qualitative study

**DOI:** 10.1002/hsr2.1655

**Published:** 2023-10-25

**Authors:** Shajwan S. Nanakali, Osama Hassan, Luisa Silva, Amani Al‐Oraibi, Jonathan Chaloner, Mayuri Gogoi, Irtiza Qureshi, Pankhuri Sahare, Manish Pareek, Kaushik Chattopadhyay, Laura B. Nellums

**Affiliations:** ^1^ Lifespan and Population Health Academic Unit, School of Medicine, Nottingham City Hospital University of Nottingham Nottingham UK; ^2^ Department of Respiratory Sciences University of Leicester Leicester UK; ^3^ College of Population Health University of New Mexico Albuquerque New Mexico USA

**Keywords:** antibiotics, antimicrobial resistance, living conditions, migrants, water, sanitation and hygiene (WASH)

## Abstract

**Background and Aims:**

Antimicrobial resistance (AMR) is among the top public health concerns around the globe. Migrants, especially forced migrants, could be at higher risk of acquiring and transmitting AMR during their journeys or in host countries. There is limited understanding regarding migrants' living conditions and the wider factors contributing to their risk of acquiring infections, and behaviors around antimicrobial use, and AMR development. In this study, we aimed to explore transit experiences, living conditions, and antibiotic use of migrants living in the United Kingdom.

**Methods:**

We conducted semistructured qualitative interviews with 27 participants and identified five themes regarding migrants' journey and their living conditions during transit and after arriving in the United Kingdom, their access to water, sanitation and hygiene (WASH), and their use of antibiotics.

**Results:**

Migrants, particularly forced migrants, experienced unfavorable living conditions, poor access to WASH, and challenges in accessing healthcare, which further contributed to health conditions like urinary and skin problems. Isolation and difficulty in accessing healthcare played significant roles in migrants' perceived need for storing and using antibiotics as a safety net.

**Conclusion:**

The findings highlight the need for coordinated and multilevel interventions to address these challenges and contribute toward tackling AMR and improving the health of this population group.

## 1 INTRODUCTION

Antimicrobial resistance (AMR) is one of the leading public health threats causing universal morbidity and mortality.^1^ The lack of quick development of new antibiotic classes together with overuse and misuse of antibiotics raises concerns around the insufficiency of the medical sector to tackle antimicrobial resistance.^2^, ^3^ Migration and international travel are thought to play important roles in the acquisition and spread of AMR.^4^, ^5^ Resistant strains are consistently associated with preventable deaths and excess costs across high‐ and low‐income countries and have been found across countries in every continent,^6^, ^7^ emphasizing the need for a global united approach in analysing and tackling AMR.^8^ It is estimated that by 2050, AMR will cause approximately 10 million deaths and costs reaching up to 100 trillion USD if effective actions are not adopted.^8^, ^9^ This emphasizes the need to tackle the human‐related factors, such as over‐consumption, over‐prescription, poor control of infectious diseases, lack of awareness of antibiotic use and AMR, increase in population size and connectivity, and the complex wider factors that contribute to facilitating this public health issue.

According to the Office of the United Nations High Commissioner for Human Rights (OHCHR), an estimated 3.6% of the human population consisting of 281 million people are considered migrants, living outside their country of birth.^10^ There may be differences in prescription and consumption rates among migrants in host countries influenced by varied expectations among patients and providers or presentation of symptoms.^11^ Evidence suggests that some groups of migrants, such as refugees and asylum seekers, are at higher risk of acquiring AMR during the migration trajectory, or in host countries due to underlying social factors like overcrowding at refugee camps or temporary facilities, disrupted access to healthcare, and poor access to water, sanitation and hygiene.^12^ The prevalence of AMR and the use of antibiotics among migrants should be targeted sensitively to avoid stigmatizing or blaming these individuals for carrying AMR to host countries.^13^ More robust research is needed to understand the drivers of antibiotic use and resistance in different migrant groups. This may in turn inform effective policies and avoid holding migrants, including refugees and asylum seekers, responsible for AMR in host countries, which may negatively affect immigration policies.^5^, ^13^

In 2021, there were an estimated six million Individuals born outside the UK living in the United Kingdom, including migrants who came for purposes of work, study, family, or seeking asylum or refuge.^14^ As of November 2022, there were 231,597 refugees, 127,421 pending asylum cases, and 5483 stateless people.^15^ Asylum seekers are provided with housing by the government. However, reports have shown these accommodations are often suboptimal with high risk of infectious disease outbreaks, such as COVID‐19. There have also been damning reports around safety and risk of disease transmission.^16^, ^17^ Funding for food, sanitation, and clothing is provided per day, however evidence suggest that this is insufficient, driving individuals to poverty and increasing the need for support from charities.^15^, ^18^

Overall, there is limited research investigating the role of wider socioenvironmental factors in relation to prescribing (and overprescribing) as well as consumption (and overconsumption) of antibiotics among migrants. This area is under‐researched, particularly in terms of qualitative research to attain migrant populations' insights on complex factors and lived experiences contributing to their antibiotic use, risk of infectious diseases and AMR. Such research is needed to target the health needs of migrants in communities and provide tailored care strategies and interventions. We aimed to explore the perspectives of migrant populations around their migration experiences, living conditions and access to WASH, and antibiotic use in the United Kingdom. This study was part of a wider mixed‐method research project entitled Examining Migration and the Epidemiology of Resistance in Groups in Europe (EMERGE), aiming to identify and explore individual, community and broader factors contributing to AMR acquisition and spread.

## 2 METHODOLOGY

We conducted a qualitative study with migrants living in the United Kingdom. This study included a diverse sample of migrants (including migrants on visas, forced migrants such as asylum seekers and refugees, and migrants with European [EU] and UK citizenships), aged 18 years and older, born outside the United Kingdom (with no limit on arrival date), and deemed to have capacity to consent. Eligible participants were recruited through collaborators such as Patient and Public Involvement and Engagement (PPIE) groups, community organizations such as Doctors of the World, and snowball sampling throughout March–July 2022. The study was also promoted through posters. Preference for interview timing and location (online using Microsoft Teams, phone call, or face‐face) were given by the participants and arranged by the research team. Purposive sampling was used to ensure the inclusion of all relevant migrant groups.^19^ Participation involved filling in a demographic questionnaire and taking part in an interview. Informed consent was obtained from all participants. The interviews lasted between 21 and 45 min, with a mean duration of 30 min. Snowball sampling was used for further recruitment, which is a recommended data collection method to include undeserved/underrepresented groups such as migrants in research.^20^

Interviews requiring translation were conducted by two researchers, the first researcher taking the lead on asking the questions, and the second translating, to increase rigor and accuracy. We carried out semistructured interviews. The interview topic guide was developed based on WHO's multicountry public awareness survey regarding antibiotic resistance and discussion with the study PPIE group.^11^, ^21^ It was piloted in the initial five interviews; the pilot interviews were included in the analysis. Recruitment was conducted alongside data analysis and guided by data saturation. Consolidated criteria for reporting qualitative research (COREQ) guidelines were followed in this paper. Each participant was given a £20 voucher after the interviews as appreciation for their time.

All interviews were recorded, transcribed and anonymised. Transcribed interviews were analysed using the thematic analysis approach described by Braun and Clarke (2006),^22^ using Nvivo (2020) software and field notes were written during/after the interviews.

## 3 RESULTS

**Figure 1:**
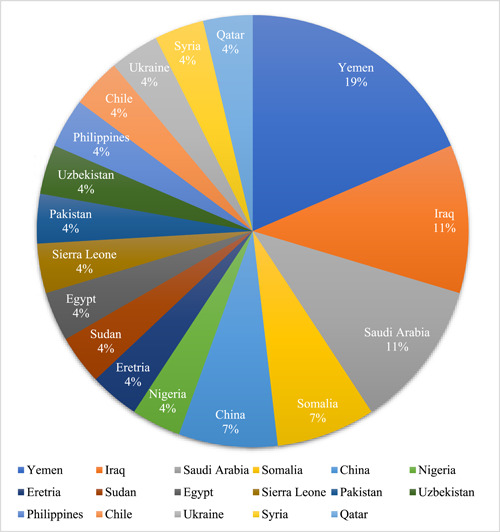
Participants countries of origin.

Twenty‐seven participants were recruited and interviewed based on data saturation. The participants came from diverse backgrounds and regions of the world (Figure 1). Participants' characteristics are presented in Table 1.

**Table 1:** Characteristics of participants (*n* = 27).

Characteristics	*N* of participants (%)
*Age*	
*Range*: 21–60 years	
*Median: 33 years*	
*Gender*	
Male	17 (63%)
Female	10 (37%)
*Immigration status*	
Visa	7 (26%)
Asylum seeker	5 (18.5%)
British citizen	4 (14.8%)
Unclear	4 (14.8%)
Refugee	3 (11%)
Leave to remain	2 (7.4%)
EU citizen	1 (3.7%)
Prefer not to answer	1 (3.7%)
*Years in the United Kingdom*	
0–2 years	14 (51.9%)
2–5 years	6 (22.2%)
5–10 years	2 (7.4%)
>10 years	5 (18.5%)
*Household*	
Single adult	14 (51.9%)
Single adult with child/children	1 (3.7%)
Married	2 (7.4%)
Married with children	4 (14.8%)
Multiple adults	4 (14.8%)
Multiple adults with child/children	1 (3.7%)
Prefer not to say	1 (3.7%)

**Figure 2:**
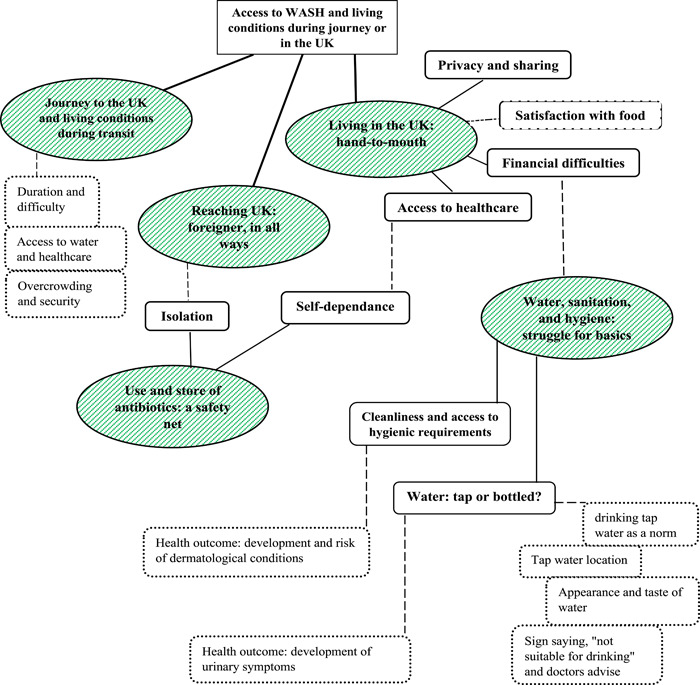
Thematic map of findings. Green circles: The main themes. Highlighted rectangles: Subthemes. Dashed lines and boxes: Related links and topics.

We identified five main themes relating to the aim of this paper: (1) Journey to the United Kingdom and living conditions during transit, (2) Reaching the United Kingdom: foreigner, in all ways, (3) Living in the United Kingdom: hand‐to‐mouth, (4) Water, sanitation, and hygiene: struggle for basics, (5) Use and store of antibiotics: a safety net. The themes and corresponding subthemes are summarized and further broken down in a thematic analysis map illustrated in Figure 2.

### 3.1 Journey to the United Kingdom and living conditions during transit

Many of the forced migrants explained their migration to the United Kingdom as long and difficult, especially when transiting several countries via different routes such as boats, airplanes, or with smugglers. As much as forced migrants were hesitant in giving details about their journey, feeling helpless, and powerless was noted when journeys were described. The negative impact of traveling also seemed to extend beyond the journey, with individuals feeling panic even upon reaching the United Kingdom.

I was in a bad situation inside the plane [where the participant hid] because I came illegally, and I also was in a critical situation. (M18, Male, Asylum seeker/Refugee)

Forced migrants described the difficult living conditions and their limited access to clean drinking water during migration. For example, one asylum seeker described dangerous situations in transiting countries, experiencing overcrowding, and a violation of the human right to safety.

The living conditions were really hard. I stayed in a prison and the conditions as mentioned were very hard and I didn't have access to clean water. I was using water bottles and they were filling them with many sources of water and these sources of water was not clean. (M17, Male, Asylum seeker/Refugee)

Some migrants mentioned that they or their family members suffered from medical conditions when traveling, putting an extra burden on their migration journey. Worsening of conditions and lack of medications were mentioned during long journeys.

I came out from (place 2) and had a tube inserted from my kidney to my bladder… My journey lasted around one month, there was a smuggler taking me… The condition was hard to get any medical intervention, so I had no choice other than being patient with this pain… I couldn't get medications when I needed that. (M13, Female, Asylum seeker/Refugee)

Two of the forced migrants with irregular trajectories also reported being admitted to hospital directly upon arrival in the United Kingdom due to the health conditions they had, and one migrant expressed gratitude: “They saved my life.” Other migrants holding visas such as students, had a more “straightforward” and “smooth” journeys to the United Kingdom with air travel.

### 3.2 Reaching the United Kingdom: Foreigner, in all ways

Participants described reaching the United Kingdom and their feelings toward a country that is different from their own. Most participants made differentiations and explained the contrasting aspects and systems between their country of origin and the United Kingdom: “Even the building, almost everything is different” (M05, Male, Visa). Some participants mentioned traveling to a new country and being a foreigner encouraged them to bring antibiotics and other medications with them, due to lack of familiarity with the host country and the health system.

The reason I bought (antibiotics) is just a very common precautious medicine that you, I think you should have, I should have like with me because when I first reach here, I was in a foreign country, I didn't know anything and at the beginning everything was not set up. I wasn't settle down. So just in case, if I get sick, I have a something near me. (M27, Female, Visa)

### 3.3 Living in the United Kingdom: Hand‐to‐mouth

#### 3.3.1 Privacy and sharing

Forced migrants explained their experiences when initially placed in hotels, and many had positive views when describing their single rooms in terms of privacy and personal space. However, some migrants had less positive experiences, and described having to share space with others in accommodations provided by the council.

Before leaving the hospital I was living in a hotel, but when I left the hospital they gave me a new place to live. It was a studio and everything was available at the studio, and everything I need, I have my own bathroom, my own kitchen, and I was comfortable living there… Currently living condition has changed, because I'm living in a shared house with three people, three ladies, and we share the bathroom, but we're trying hard to keep the cleaning as much as possible in. (M13, Female, Asylum seeker/Refugee)

#### 3.3.2 Financial difficulties

Migrants described financial difficulties and the impact these had on their living conditions, health, and ability to afford basic needs. Inability to afford medications was mentioned as a barrier to following up healthcare professionals' advice fully by one of the migrants as follows:

They told me to take one tablet every week for six weeks, and then I have to buy the medication by myself…but I didn't have money at that time. (M14, Male, Asylum seeker/Refugee)

Migrants on visas also mentioned financial difficulties in the United Kingdom and the need to work hard and live with necessities. *“*It was very difficult because it basically it was a struggling phase of our life …financially we were hand‐to‐mouth.” (M26, Female, Leave to remain)

Some participants also explained how affording medications in their countries of origin is easier and how that acts as a driver to bringing in medications with them when coming to the United Kingdom to avoid the cost.

#### 3.3.3 Access to healthcare

One of the key challenges highlighted by migrants was access to healthcare. A number of migrants expressed frustration and long waiting periods to see a doctor. Some forced migrants mentioned seeking/getting help from third parties like refugee organizations and teachers. The third parties help seemed to have a positive impact in delivering medications and assisting migrants in accessing hospitals more quickly.

To take an appointment for my operation I struggled as well because they gave me a very far appointment and I started to develop more symptoms, like, vomiting. I felt unwell and then my teacher helped me and she called the NHS and advised me to go to the hospital directly. When I went to the emergency room directly when they saw my condition they were, like, they did the operation right away. (M11, Male, Asylum seeker/Refugee)

Migrants also shared other routes they took to get healthcare or help for their conditions when they struggled in getting appointments, such as taking previously prescribed medications or having to go to walk‐in clinics. Difficulty in getting appointments to access healthcare was mentioned and described as a challenge by all migrant groups. The long waiting time to access healthcare was considered odd to participants and caused confusion when compared with accessing healthcare in their countries of origin, especially emergency services.

My neighbour here upstairs, a lady, she was suffering from back pain or something. She was crying like half a day here in the reception trying to reach NHS, like a half day of crying from the pain, and nobody came. It was like shocked because I believe UK is one of the best countries and it was like they have service here supposed to be like high standard. Somebody is suffering and is waiting for this long time. (M16, Male, Asylum seeker/Refugee)

Some migrants also described difficulties in communication, and linguistic assistance they received from primary healthcare providers, such as using online translator applications. In cases where interpreters were provided, migrants seemed more satisfied with communication and their experience with healthcare. However, challenges with appointing and trusting interpreters were also mentioned.

I communicate with them in English and sometimes he [the GP] helps me by using Google Translate, because it's difficult to arrange an interpreter and it takes time, and I have to change the appointment. It sometimes takes one week to get an interpreter. (M20, Male, Asylum seeker/Refugee)

Sometimes people don't trust on the interpreters… So I don't know whether what I'm saying the interpreter is communicating correctly to that person. (M26, Female, Leave to remain)

One migrant explained the role of linguistic barriers and miscommunication in causing insecurity and not asking for healthcare or sharing concerns with others as follows.

There wasn't a really good experience, so I just try to not have many communication with them… I try to express myself and I couldn't, so were too many things. that's why I never say anything there. (M01, Female, Visa)

### 3.4 Water, sanitation, and hygiene: Struggle for basics

#### 3.4.1 Water: Tap or bottled?

Water was frequently mentioned in the interviews. Forced migrants explained having to buy bottled water from markets. Some migrants seemed unsure about drinking water from the tap in the host facilities. “Once I came I was shocked when I asked him where I can drink…He say you just drink from the tap” (M16, Male, Asylum seeker/Refugee). Location of the tap water also seemed to play a role in migrants not wanting to drink it, for example with the only source of tap water being located in the bathroom of the hotel accommodations.

I bought water from outside, drinking water because the tap water is in the bathroom, and I can't use the sink to drink water so I'm buying my water from outside. (M12, Female, Asylum seeker/Refugee)

One migrant explained the appearance and taste of water not being acceptable or clean to him, which led him to avoiding the tap water. Buying bottled water added an extra financial burden on the migrants. *“*I sometimes bring water from the market or sometimes drink from the regular tap water depending on financial conditions.” (M13, Female, Asylum seeker/Refugee). Participants also described the water was affecting their health. *“*I used to use the tap water but I developed kind of, urinary symptoms and I then stopped using the tap water and have to buy water from the market.” (M11, Male, Asylum seeker/Refugee). Participants also mentioned being told by accommodation managers or doctors to avoid drinking from the tap *“*They put a sign that the tap water is not usable to drink.” (M20, Male, Asylum seeker/Refugee).

#### 3.4.2 Cleanliness and access to hygienic requirements

There was a notable dissatisfaction with cleaning conditions in the host facilities. Some participants mentioned varying frequencies of cleaning services provided by different hotel facilities, with some providing more frequent cleaning services. Others mentioned being self‐dependent and doing their own cleaning, which extended to their placement in houses. Poor conditions such as fungus/mould were mentioned as well.

Some participants described their access to hygienic requirements such as towels and shaving kits, and how they struggled accessing or affording them and having to share them: *“*We were sharing the shaving materials and sharing it with other people.” (M14, Male, Asylum seeker/Refugee). Development of dermatological conditions was mentioned among participants. Some mentioned having skin reactions without attributing their health condition to their living conditions, while others directly talked about their skin conditions as an outcome of the poor cleanliness and sharing personal hygienic requirements with others. *“*When I came to the UK and stayed in the hotel after two weeks I developed a severe allergic reaction.” (M12, Female, Asylum seeker/Refugee)

### 3.5 Use and store of antibiotics: A safety net

Most migrants were aware that getting antibiotics in the United Kingdom requires a prescription. The majority of the migrant participants expressed that they don't normally use antibiotics without a prescription, and some even expressed their “fear” of antibiotics and one even mentioned “trashing” them whenever they felt better due to the consequences antibiotics may have. Seven of the migrants talked about using or storing antibiotics without a prescription, and gave insights explaining why they needed to, based on their experiences and living conditions. The subthemes and quotes are presented in Table 2.

**Table 2:** Subthemes and supporting quotes of theme 5.

Subtheme	Quotes
3.5.1 Isolation. Migrants, and especially forced migrants, described how social isolation, lack of activities in the facilities and being lonely impacted their perspectives and well‐being overall. This sense of isolation also influenced decisions around their health. For example, one of the migrants described storing and taking antibiotics as they felt they had no other choice, particularly because they didn't have support or someone to help if they got sick.	“One of the reasons that I takes them (antibiotics) is that I am alone and it is really difficult to be alone and sick here. I don't know where to go or what I do if something bad happens to me here. So I don't want my case or my condition to get worse that's why I take antibiotics.” (M18, Male, Asylum seeker/Refugee)
3.5.2 Self‐dependence. Asking for antibiotics, storing them or even taking them without prescription were noted as routes migrants adopted to be more dependent on themselves and less on the healthcare system, or even friends and relatives. Migrants also mentioned being advised by others to bring in antibiotics with them as they are hard to get in the United Kingdom. Some migrants mentioned the antibiotics they had were unopened, not used or even expired, emphasizing that the antibiotics were kept to give the feeling of security.	*“*My relatives send some [antibiotics] over. But this is just for the emergency use, if I can't access the GP in the same day. They are mostly expired and never been used because this is just, you have something in the pocket, you feel safe.” (M24, Male, British citizenship) “It's antibiotics, just in case. Just in case, because sometimes you don't know what might happen tomorrow, for example you need but you can't take appointment with a doctor, but you need straight away. What to do? Just in case you know. I prefer if I have something and maybe in the future, I don't need it but I have it.” (M25, Female, EU citizenship)

## 4 DISCUSSION

Previous work on migration has shown that forced migrants may be exposed to abuse, exploitation, violence, and poor living conditions with limited access to basic necessities during migration.^23^, ^24^, ^25^ The findings of this study have shed light on the implications for the use of antibiotics and AMR in migrant groups. They support previous studies in showing the exposure to violence and poor conditions, and risk factors for poor health outcomes and spread of infection including overcrowding, and limited access to basic necessities, clean water, or healthcare that particularly forced migrants experience during long transits.^23^, ^24^, ^25^

For asylum seekers, the Home Office in the United Kingdom provides initial temporary accommodation (e.g., in hotels) until they are assigned a more permanent accommodation (usually shared). However lately, there have been concerns that time of stay in initial accommodations is reaching 6 months to 1 year, with inadequate access to basic essentials.^26^ In this study, participants expressed mixed experiences with hotel stays. Housing problems such as mould, poor conditions and cleanliness, and a lack of hygienic necessities were identified, all of which have the potential to contribute to the spread of infection. Similar findings of sharing and lack of privacy, unhygienic facilities and dissatisfaction with food quality were also reported as post migration stressors in a qualitative explorative study conducted in Belgium.^27^ Housing is considered as a social determinant of health, and conditions like overcrowding, unsafe water, humidity, and mould can directly contribute to risk of communicable diseases or antibiotic use.^28^ For example, a mixed‐methods systematic review found significant housing problems associated with worsening physical health and quality of life in refugees in both camps and resettlement countries.^28^ Negative health impacts due to poor housing conditions and the lack of control migrants may have over this have been explored in previous qualitative research.^29^ Overall, migrants, and especially more vulnerable groups of forced migrants, may have higher risks of communicable diseases and be more likely to be prescribed antibiotics. For example, in primary care wards in initial refugee reception centers in Germany, up to 75% of antibiotic prescribing was found to be inappropriate (e.g., where antibiotics weren't indicated).^30^

Research has also shown that migrants are at increased risk of financial exclusion in the United Kingdom, which presents barriers to accessing or affording services.^31^, ^32^ This study adds on direct perceptions of migrants' difficulties in describing the cost of medications in the United Kingdom as a driver for them to bring back medications, including antibiotics from their countries of origin. Others explained their struggle to afford basic necessities, such as hygienic materials and water.

Regarding accessing healthcare in host countries, this study found that all migrant groups regardless of status, reported challenges in accessing health, aligning with previous evidence that migrants experience both language and trust barriers.^33^ Frustration and confusion were also found among the participants when talking about long waiting times. In some cases, missing appointments and miscommunications were also mentioned, highlighting limited knowledge regarding differences in accessing primary, secondary and tertiary care, especially for migrants who were newly arrived. These findings provide an update of previous findings, and reflect themes reported by a qualitative study examining asylum seekers' and refugees' access to primary care in the United Kingdom, where lack of awareness of how the NHS functions was reported.^34^

One of the five main objectives of the World Health Organisation's (WHO's) global action plan to address AMR is “to reduce the incidence of infection through effective sanitation, hygiene and infection prevention measures.”^35^ Nevertheless, preventing or controlling spread of infectious diseases is mandatory to decrease the requirement of antimicrobials.^36^ Large inequalities in accessing adequate water, sanitation, and hygiene are identified among refugee camps and settlements.^37^ Excess infectious cases and high burden of disease have been attributed to improper WASH in sub‐Saharan refugee camps.^38^ Furthermore, previous studies have found dermatological conditions such as scabies and skin infections, as common morbidities among migrants crossing the Mediterranean Sea, which are presumed to be related to poor living conditions along the journey or upon reaching host countries.^39^, ^40^ This study adds knowledge of migrants' experiences of overcrowding, and poor WASH, in some cases resulting in the development of skin problems after reaching the United Kingdom and during their stay at host facilities. Poor cleanliness, limited access to and sharing of personal hygiene materials described by migrants, could play an important role in putting these groups at risk of skin conditions and spread of infection, increasing the need to seek healthcare.

The findings of water‐related issues in the United Kingdom reported in this study are novel and detailed in explaining experiences in accessing clean drinking water in the United Kingdom from migrants' perspectives. Though drinking water from the tap is considered normal in the United Kingdom, it may not be acceptable for many migrants due to different norms from their own backgrounds, as a great number of countries don't have potable tap water. In addition, in some cultures drinking from bathroom may not be considered acceptable, normal, or clean, explaining hesitancy of migrants to drink directly from the tap in the hotel accommodations. Beyond these perceptions around tap water among migrants, the suitability of tap water was also brought into question, as some migrants reported it was unclean (discolored or with debris), and that they were advised to avoid drinking from the tap in some facilities.

With regard to antibiotics, our findings highlighted that the majority of migrants rely only on prescriptions to get antibiotics in the United Kingdom. A previous study in the Netherlands concluded that newly arrived migrants are more likely to expect antibiotics from doctors, though they may not exert pressure for antibiotics prescriptions.^41^ In this study, it was found that migrants' expectations are greatly due to what they are used to in their countries of origin, with differences across systems. In cases where antibiotics were brought from countries of origin or obtained from friends and family and stored, a sense of safety was described by migrants. For example, being isolated made them scared of getting sick away from family or friends and led them to rely more on antibiotics to avoid getting worse. Social isolation of migrants has also been highlighted in previous studies.^42^ Antibiotics were also stored as an alternative plan or in case accessing healthcare was challenging in the future. In support of these findings, a previous study conducted in 2016 interviewing newly arrived migrants, explored that previous experiences with healthcare, uncertainty and moving to a new country impacted migrants' views regarding use of antibiotics in the United Kingdom.^11^ The store of left‐over antibiotics for later use is also reported in a qualitative study among British citizens, with only a minority reporting storing antibiotics. The respondents were young adults from deprived areas,^43^ raising a hypothesis that more excluded or marginalized groups may perceive a feeling of need to store antibiotics.

There is a major need to support knowledge about access to different services and healthcare in the United Kingdom for all migrant groups, especially new arrivals. This includes information through appropriate means/languages regarding antibiotics, and AMR as a global issue. More studies are needed specifically in forced migrant groups in cooperation with international organisations such as the (WHO). Studies looking at the prevalence of skin and urinary tract conditions through medical assessment and laboratory confirmation are needed in forced migrants. In addition, adequate interpretation services should be provided alongside registration and access to healthcare. Difficulty in appointing an interpreter should not add extra waiting time to see a doctor because it may lead to worsening health conditions and drive migrants to seek help from nonhealthcare professionals or take medications including antibiotics without proper consultations. Water fountains or standing water dispensers, stable adequate cleaning services across hotels, with adequate supply of personal hygienic needs, adequate quality and quantity of food, together with provision of interactive activities to promote social inclusion are essential to support migrants' well‐being.

## 5 CONCLUSION

This study highlights social and environmental health inequalities in terms of poor living conditions and access to WASH faced by migrant groups generally and forced migrants particularly. The findings emphasize the wider factors that impacted migrants' health needs and perceptions regarding storing and using antibiotics. The majority reported how hard it is to get antibiotics without a prescription and not using them unless with a doctor's advice and prescription. Some migrants explained how storing antibiotics gave them assurance due to their limited social support, and that storing antibiotics was a back‐up plan for possible future difficulties in getting appointments or accessing care. The findings highlight the need for more research and multilevel interventions addressing these challenges (that impact migrants' risk of infections and antibiotic use) to improve the health of this population group and tackle AMR. We advocate that decision makers should seek to develop control and prevention strategies informed by the study findings.

## AUTHOR CONTRIBUTIONS

**Shajwan S. Nanakali**: Conceptualization; data curation; formal analysis; investigation; writing—original draft. **Osama Hassan**: Data curation; investigation. **Luisa Silva**: Data curation; investigation; writing—review & editing. **Amani Al‐Oraibi**: Data curation; investigation; writing—review & editing. **Jonathan Chaloner**: Data curation; investigation; writing—review & editing. **Mayuri Gogoi**: Writing—review & editing. **Irtiza Qureshi**: Writing—review & editing. **Pankhuri Sahare**: Data curation. **Manish Pareek**: Writing—review & editing. **Kaushik Chattopadhyay**: Supervision; writing—review & editing. **Laura B. Nellums**: Conceptualization; data curation; funding acquisition; methodology; supervision; writing—review & editing. All authors have read and approved the final version of the manuscript.

## CONFLICT OF INTEREST STATEMENT

L. B. N. is supported by an Academy of Medical Sciences Springboard Award (SBF005/1047). M. P. reports grants from Sanofi and Gilead outside the current work and has received consulting fees from QIAGEN and Pfizer. The remaining authors declare no conflict of interest.

## ETHICS STATEMENT

Ethical approval for the EMERGE project was granted by the Faculty of Medicine and Health Sciences University of Nottingham, (reference no. FHMS 411‐1121).

## TRANSPARENCY STATEMENT

The lead author Shajwan S. Nanakali affirms that this manuscript is an honest, accurate, and transparent account of the study being reported; that no important aspects of the study have been omitted; and that any discrepancies from the study as planned (and, if relevant, registered) have been explained.

## Data Availability

All data is stored on the University of Nottingham secure server for 10 years, in line with the University of Nottingham research data management policy. Due to participants' privacy protection supporting data is not available publicly. Anonymised data can be provided upon request. The corresponding author had full access to all of the data in this study and takes complete responsibility for the integrity of the data and the accuracy of the data analysis.
